# *In vitro* efficacy of potentiated egg yolk powder against *Campylobacter jejuni* does not correlate with *in vitro* efficacy

**DOI:** 10.1371/journal.pone.0212946

**Published:** 2019-03-07

**Authors:** Amina Soumaila Garba, Alexandre Thibodeau, Audrey Perron, Sylvette Laurent-Lewandowski, Ann Letellier, Philippe Fravalo

**Affiliations:** 1 Chaire de Recherche industrielle du CRSNG en salubrité des viandes, Faculté de médecine vétérinaire, Université de Montréal, Saint-Hyacinthe, QC, Canada; 2 Groupe de recherche et d'enseignement en salubrité alimentaire, Faculté de médecine vétérinaire, Université de Montréal, Saint-Hyacinthe, QC, Canada; 3 Centre de recherche en infectiologie porcine et avicole, Faculté de médecine vétérinaire, Université de Montréal, Saint-Hyacinthe, QC, Canada; United States Department of Agriculture, Agricultural Research Service, UNITED STATES

## Abstract

*Campylobacter jejuni* is a zoonotic agent responsible for the foodborne gastroenteritis campylobacteriosis. Control of *C*. *jejuni* load in the poultry primary production is recognized as an avenue to reduce human exposure to the pathogen. As for now, no commercially applicable control methods exist at the farm. Several studies tested egg yolk powders, potentiated or not against *C*. *jejuni*, as feed additives for chicken and suggested that the quantity and quality of the antibodies presence in the yolk are determinant factors for the full success of this approach. Unfortunately, data from these studies inconsistently showed a reduction of cecal *C*. *jejuni* carriage. Our first goal wwas to characterize (quantification by ELISA, agglutination test, bacterial antigen recognition profiles by Western blot, bactericidal effect by serum killing assays and *C*. *jejuni* mobility by soft agar migation) the antibodies extracted from egg yolk powders originating from different egg production protocols. Secondly, these powders were microencapsulated and recharacterized. Finally the protected powders were tested as a feed additive to destabilize *C*. *jejuni* colonization in an *in vivo* assay. Despite the *in vitro* results indicating the ability of the egg yolk powders to recognize *Campylobacter* and potentially alter its colonization of the chicken caecum, these results were not confirmed in the *in vivo* trial despite that specific caecal IgY directed toward *Campylobacter* were detected in the groups receiving the protected powders. More research is needed on *Campylobacter* in order to effectively control this pathogen at the farm.

## Introduction

Currently, campylobacteriosis is the most important bacterial foodborne disease in the world, evaluated to induce 7.5 million years of disability-adjusted life years (DALY) [1]. The economic costs associated with campylobacteriosis are tremendous [2]. Serious complications such as Guillain-Barré syndrome, reactive arthritis, and irritable bowel syndrome can be severe consequences of campylobacteriosis [3]. Handling or consumption of raw or undercooked poultry meat have been identified and regularly confirmed as a major source of human contamination by *C*. *jejuni*, the etiological agent of this foodborne disease [4, 5]. *C*. *jejuni* is a bacterium of the digestive tract acting like a commensal and thus being asymptomatically carried, up to 10^8^ CFU / g [6]. This bacterium currently does not multiply in food during processing or storage, stressing the importance of on farm approach to reduce the amount of *C*. *jejuni* reaching the consumer [7]. Quantitative risk assessments indicated that a 3 log10 reduction of *C*. *jejuni* in the intestines of chickens or a reduction of 2 log10 on the carcass would efficiently reduce public health risks [8, 9].

Various control measures have been tested at the farm level to reduce the colonization of poultry by *C*. *jejuni* but no consistently effective control strategy is yet commercially available [10–13]. In conventional chicken rearing conditions, *C*. *jejuni* is usually undectable during the first 2 or 3 weeks of life [14]. This lag phase had been attributed, in part, to the presence of maternal IgY antibodies transmitted to the chicks via the egg yolk [15, 16]. Using a constant supply of antibodies during the whole rearing period therefore appears as an elegant option for controlling *C*. *jejuni* chicken colonization at the farm.

An early study suggested a protective effect of oral inoculation of chickens when the *C*. *jejuni* inoculum was pre-incubated with IgYs [17]. The authors reported a significant reduction in the average number of *C*. *jejuni* per gram of cecal contents, but these reductions were brief as the counts of *C*. *jejuni* returned to the control condition levels after 2 or 3 days. The question of prolonging the action of protective antibodies was therefore raised. Kassaify and Mine reported that a prophylactic treatment with 10% egg yolk powder (EYP) for 4 weeks before infection resulted in 3–4 log reduction of *C*. *jejuni* fecal counts 7 days post inoculation, in birds of 22–24 weeks of age [18]. More recently, Hermans et al observed that the use of a potentiated egg yolk powder (PEYP) incorporated at 5% in the diet for 4 days before infection, reduced the horizontal transmission among birds in a seeder model and lead to a drastic reduction of 4.4–6.7 log10 CFU in the seeders 3 days post inoculation [19]. Conversely, Paul and Al-Adwani reported that a long-term prophylactic treatment (until 3 weeks of age) with 10% in-feed of EYP derived from hyper-immunized hens or non-immunized hens was not able to significantly reduce cecal counts of *C*. *jejuni*, 7 days post inoculation [20].

It therefore appears that the anti-*Campylobacter* effects of EYP are inconstant. It is difficult to attribute the effects to the whole EYP or specifically to the antibodies they contain. Moreover, these studies did not characterize the antibodies contained in the egg yolks powders and did not assess the benefits of protecting the EYP from degradation during the digestive transit. Protection could improve the efficiency of the EYP delivery and reduce the amount of EYP required to destabilize *C*. *jejuni* colonization. Different encapsulation modes exist for EYP, such as dehydration by spray drying, spray cooling, spray chilling or lyophilization [21]. Encapsulation methods often uses natural embedding materials such as sugar, proteins, lipids, and synthetic or modified polymers [22] to protect a molecule of interest from digestive degradation. Lipid encapsulation, already successfully applied for other food additives, appears as a simple and cost-effective solution for implementation of a protection strategy [23].

In the present study, we aimed at improving the use of EYP for the control of *C*. *jejuni* broiler colonization. Our specific goals were 1) to characterize the antibodies extracted from EYP originating from eggs produced using different protocols, 2) to assess the impact of encapsulation on the EYP ability to recognized different *C*. *jejuni* strains and 3) to test the efficiency of these different EYP in mitigating chicken colonization by *C*. *jejuni*.

## Materials and methods

### *Campylobacter jejuni* strains

*Campylobacter jejuni* strains A2008a, B2008c, G2008b [24] and reference strain RM1221 are referred to Homologous (used to potentiate the egg yolks) whereas strains 81116 and ATCC 700819ATCC 700819 are referred to Heterologous since they were not used to potentiated the yolks and therefore give a broader indication on the recognition ability of bacteria from the genus *Campylobacter* by EYP and by protected EYP.

### Preparation of egg-yolk powders and encapsulation

Egg yolks were collected from forty specific pathogen free (SPF) White Leghorn hens divided into four groups: 1**-** control, *i*.*e*. not in contact with *C*. *jejuni* (NI), 2- orally challenged with a live *C*. *jejuni* strains mix (OI), 3- subcutaneously injected with outer membrane proteins extracted from the same *C*. *jejuni* strains (OMP), and 4- subcutaneously injected with the same mix of formalin-killed *C*. *jejuni* strains (KB). Potentiated egg yolks production and characterization of IgYs recovered are as described before [25].

Fresh egg yolks were converted to powder by spray drying (Niro Atomizer, Copenhagen, Denmark) at Agriculture and Agri-Food Canada Food Research and Development Centre (FRDC; Saint-Hyacinthe, QC, Canada). The temperature in the dryer was set to 183°C and the outlet temperature was 75°C. The resulting egg yolk powders (EYP) were separately collected according to the IgY production protocols and kept at 4°C. The powders were further encapsulated in a lipid matrix by spray cooling. The resulting encapsulated egg yolk powders (EEYP) were stored at 4°C until use.

### IgYs extraction

The EYP IgY extraction was done as described elsewhere [26], with some modifications. Briefly, 9 mL of sterile PBS and 3 g of EYP were vigorously mixed and an equal volume of chloroform added. The mixture was briefly homogenized, centrifuged at 4 000 x g for 30 min and the water-soluble fraction, which contained the IgYs, was collected.

Aliquots of each EEYP were frozen at -80°C overnight and mechanically broken using a clean mortar and pestle. A 3 gr fraction was used for EYP IgY extraction, with the addition of silica beads (MP Biomedicals, Solon, OH, USA) and the use of a FastPrep-24 Tissue and Cell Homogenizer (MP Biomedicals,) set at 6.0 m/s for 30 s before the chloroform extraction in order to maximize the release of IgYs.

### Agglutination test

For agglutination tests, Homologous and Heterologous strains were individually grown on modified charcoal-cefoperazone-deoxycholate agar (mCCDA) (Innovation Diagnostics, Saint-Eustache, Canada), supplemented with Cefoperazone and Amphotericin B (Innovation Diagnostics) at 42°C for 24 h in BD Campy*Gen* gazpak system (Fisher Scientific, Ottawa, Canada) for microaerobic growth conditions (80% N_2_, 10% CO_2_, 5% H_2_, and 5% O_2_). Individual strains were suspended in Phosphate Buffer Saline (PBS) (Fisher Scientific) to reach a 1.0 Optical Density (OD) measured at 600 nm and 200 μl of the suspension were transferred in a well of an agglutination glass plate. Afterwards, 200 μL of IgY extracts were added and the suspension was agitated. Agglutination was recorded when it appeared before a maximum incubation period of 5 min. Controls consisted of 200 μL of PBS for auto-agglutination of the strains and 200 μL of IgYs from NI extracts to assess the role of potential non-specific antibody presence on agglutination.

### *Campylobacter jejuni* total protein extractions

*Campylobacter jejuni* Homologous and Heterologous strains were used to inoculate mCCDA and incubated as described above. Bacterial cultures were individually harvested, suspended in 6 mL of Brucella Broth (Innovation Diagnostics) and centrifuged at 3 000 x g for 15 min at 4°C. The pellet was suspended in 2 mL of PBS. After sonication (5 pulses, 0.4 watt, 30 sec ON/60 sec OFF each), the suspension was centrifuged at 10 000 x g for 10 min to remove cellular debris. The total proteins were recovered in the supernatants and stored in aliquots at -20°C. The protein concentration was determined using the Pierce BCA Protein Assay Kit (Fisher Scientific).

### Total or anti-*C*. *jejuni* IgYs quantification by ELISA

Quantification of total and anti-*C*. *jejuni* IgY levels in EYP and EEYP extracts was done by enzyme-linked immunosorbent assay (ELISA), using a Chicken IgG ELISA Quantitation Set (Bethyl Laboratories, Montgomery, TX, USA). To quantify total IgYs, flat-bottomed 96-well polystyrene plates (Fisher Scientific) were coated with goat anti-chicken IgG antibodies, according to the manufacturer’s recommendations (1μL of antibodies in 100μL of coating buffer (0.05M Carbonate-bicarbonate [pH 9.6])). To quantify anti-*C*. *jejuni* IgYs, plates were coated with a suspension of the mix of the Homologous strains total protein extracts (25μg/well) in coating buffer. EYP or EEYP extracts were used diluted 1:80 000 in Sample/Conjugate Diluent (50 mM Tris, 0.14 M NaCl, 5% milk, 0.05% Tween 20) to quantify total IgYs and 1:10 000 for anti-*C*. *jejuni* IgYs.

Quantifications were based on a standard curve that was prepared according to the Chicken IgG ELISA Quantitation Set protocol (Bethyl Laboratories). Duplicate and triplicate wells were used for standards and samples, respectively.

HRP Conjugated Chicken IgG-Fc Detection Antibody (Bethyl Laboratories) diluted 1:40 000 in the Sample/Conjugate Diluent and TMB (3, 3′, 5, 5′-Tetramethylbenzidine) (Bethyl Laboratories) were successively added for revelation. The reaction was stopped by adding 100 μl of the ELISA Stop Solution (0.18 M H_2_SO_4_) (Bethyl Laboratories) and absorbance at 450 nm values of individual wells were measured by an ELISA Universal Microplate Reader "EL 800" (Bio-Tek Instruments Inc., Winooski, VT, USA) and analyzed with the KC junior software (Bio-Tek Instruments Inc.).

For EEYP, powder/encapsulation matrices ratio were considered when calculating the final concentration and assessing the effect on embedding on the original IgY concentrations.

### SDS-PAGE and immunoblotting

Total protein (40 μg/well) extracts from each *C*. *jejuni* sonicated strains were boiled in the sample loading buffer (62.5 mM Tris-HCl [pH 6.8], 10% glycerol, 3% SDS, 5% *β-*mercaptoéthanol, 0.01% bromophenol blue) at 100°C for 5 min and were separated in a 10% SDS-polyacrylamide gel electrophoresis (SDS-PAGE) with a 4.5% stacking gel. The gel was run at 100 V for 30 min immediately followed by 200 V for 2h in the running buffer (25mM Tris, 0.2M glycine, 0.1% SDS). The separated proteins were transferred onto polyvinyl difluoride (PVDF) membranes (Bio-Rad Laboratories, Mississauga, ON, Canada) using the transfer buffer (25 mM Tris [pH 8.3], 192 mM glycine, 20% methanol) at 100 V for 1h at 4°C in a Mini Trans*-*Blot cell*s*, (Bio-Rad). The membranes were blocked for 1h with a buffer (10 mM Tris [pH 7.4], 0.15M NaCl) containing 2% of skim milk powder and incubated with the IgYs extracts: two dilutions were performed for EYP extracts: 1:150 (NI–OI) and 1:300 (OMP—KB) and two dilutions were also performed for EEYP extracts: 1:75 (NI–OI) and 1:150 (OMP—KB). The membranes were washed 5 times 10 min in the wash solution (10 mM Tris, pH 7.4, 0.15M NaCl) and subsequently incubated with a peroxidase-conjugated goat anti-chicken- IgG (Bethyl Laboratories) at a dilution of 1:75 000 in Sample/Conjugate Diluent for 1h at 25°C. Finally, after 5 washes, membranes were developed by incubation in 30 mg of 4-chloro-1-naphtol (Fisher Scientific) containing 30 μl H_2_O_2_ (Fisher Scientific) and stopped in 10 ml of methanol (Fisher Scientific).

### Motility assay

Homologous and Heterologous strains grown on mCCDA were individually suspended to obtain an optical density of 1.00 at 600 nm. Motility assays were performed in Brucella Broth Medium supplemented with 0.4% Agar (Innovation Diagnostic) and with EYP or EEYP IgY extracts (final ratio 1:100).

A 50 μL volume of bacteria was pre-incubated with 50 μl of IgY for 3 min at 42°C and 10 μL was dropped on the surface of the semi-solid medium. Motility plates were incubated 24h at 42°C under microaerobic conditions. The migration/growth diameter was measured. For each strain and antibody extracts combination, three independent trials were carried.

### Bactericidal assay

The bactericidal assay was performed in sterile microcentrifuge tubes, on the Homologous strains in contact with EYP extracts from the OMP and KB groups. Each reaction contained 112 μL of diluted EYP (10% wt/vol) in Brain Heart Infusion Broth (BHI) (Innovation Diagnostic), 112 μL of serum from *C*. *jejuni*-negative chickens [23] and 25μL of bacteria suspended in BHI (OD_600_ = 1). Bacteria incubated with complement inactivated serum and EYP were used. Assays were incubated for 1h at 37°C. After incubation, 100 μL of the suspension was plated on Tryptic Soya Agar (TSA) containing 5% (v/v) of sheep’s blood (Fisher Scientific). After 48 h of incubation at 42°C under microaerobic conditions, colonies were numerated and bactericidal activity was expressed as the reduction percentage of the number of live *C*. *jejuni* after incubation compared to the control. Additional controls were included to assess specificity of the assay: bacteria + chicken serum without EYP, bacteria and diluted EYP without serum and bacteria only.

### *In vivo* assay

*In vivo* assay was performed with the approval of the Comité d’éthique de l’utilisation des animaux (CÉUA) of the Faculty of Veterinary Medicine of the Université de Montréal (accreditation number Rech-1740). Day-old chicks (n = 96) were distributed into two rooms. The first one contained eight groups (A1 to D1 and A2 to D2) of eight birds and the second housed four (A3 to D3) groups of eight birds. At day 0, each group received *ad libitum* a standard mash feed containing 5% of the different encapsulated EEYP. Control groups (A1, A2 and A3) received the empty encapsulation matrix, groups B1, B2 and B3 received the NI EEYP, groups C1, C2 and C3 received OI EEYP and groups D1, D2 and D3 received a 1/1 mix of OMP and KB EEYPs. On day 12, freshly excreted feces were collected to confirm the absence of *C*. *jejuni*. On day 14, the birds in the first room (groups A1 to D1 and A2 to D2) were inoculated orally with 2 x 10^3^ total CFU per bird of an equally concentrated mix of the Homologous strains while the birds in the second room (groups A3 to D3) were inoculated with 3 x 10^3^ CFU per bird of an equally concentrated mix of the Heterologous strains. Inoculum concentrations were determined *ad posteriori* by enumeration. On day one post-inoculation (D1 PI), D3 PI, D5 PI and D7 PI, three 5g samples of fresh feces were collected to follow the evolution of *C*. *jejuni* excretion. On day D7 PI, the birds were euthanized and fresh caecal contents were collected to enumerate the *C*. *jejuni* caecal populations and to determine the concentrations of anti-*C*. *jejuni* antibodies. Antibodies were recovered from 500 mg of caecal content as describe previously.

### Excreted and cecal *Campylobacter jejuni* numeration

Caecal contents and fresh fecal samples were diluted 1:9 (w/v) in Buffered Peptone Water (Innovation Diagnostic). After homogenization, 10-fold dilution series were made and 100μL of each dilution were plated on mCCDA. After 48 h incubation at 42°C under microaerobic conditions, colonies were enumerated.

### Statistics

Data were analyzed with GraphPad Prism 6 software (GraphPad Software, La Jolla, CA, USA). The significance level α was set at 0.05 except otherwise specified. A one-way analysis of variance (ANOVA) followed by Tukey's post hoc test was carried out to compare the means of IgY concentrations for ELISA tests. Otherwise, a Kruskall Wallis test, followed by Dunn's post hoc test was carried out to compare growth diameters, the migration average ratios for motility and the means of log CFU counts in *in vivo* assays. A T test was done for assessing the bactericidal effect.

## Results

### Agglutination test

The antibodies contained in both EYP and EEYP corresponding to groups OI, OMP and KB promoted agglutination of the Homologous and the Heterologous strains. As expected, the agglutination appeared faster and were easier to record with OMP, KB (Table 1) and with the IgY purified from EYP as compared to EEYP.

**Table 1:** Agglutination against Homologous and Heterologous *C*. *jejuni* strains for EYP and EEYP extracts.

		NI		OI		OMP		KB	
		EYP	EEYP	EYP	EEYP	EYP	EEYP	EYP	EEYP
**Homologous**	**G2008b**	**-**	**-**	**	*	***	**	***	**
	**A2008a**	**-**	**-**	**	*	***	**	***	**
	**B2008c**	**-**	**-**	**	*	***	**	***	**
	**RM1221**	**-**	**-**	**	*	***	**	***	**
**Heterologous**	**ATCC 700819**	**-**	**-**	**	*	***	**	***	**
	**81116**	**-**	**-**	**	*	***	**	***	**

The agglutination observed with EYP and EEYP extracts against Homologous (G2008b, A2008a, B2008b and RM1221) or Heterologous (81116, ATCC 700819) *C*. *jejuni* strains. Symbols ***, **, * and–indicate that agglutination was observed in less than 5 min, 3 min, 1 min, or no agglutination observed, respectively. NI: control; OI: orally challenged; OMP: subcutaneously injected with outer membrane proteins; KB: subcutaneously injected with formalin-killed bacteria.

### Quantification of total and anti-*C*. *jejuni* IgY in EYP and EEYP

**Fig 1:**
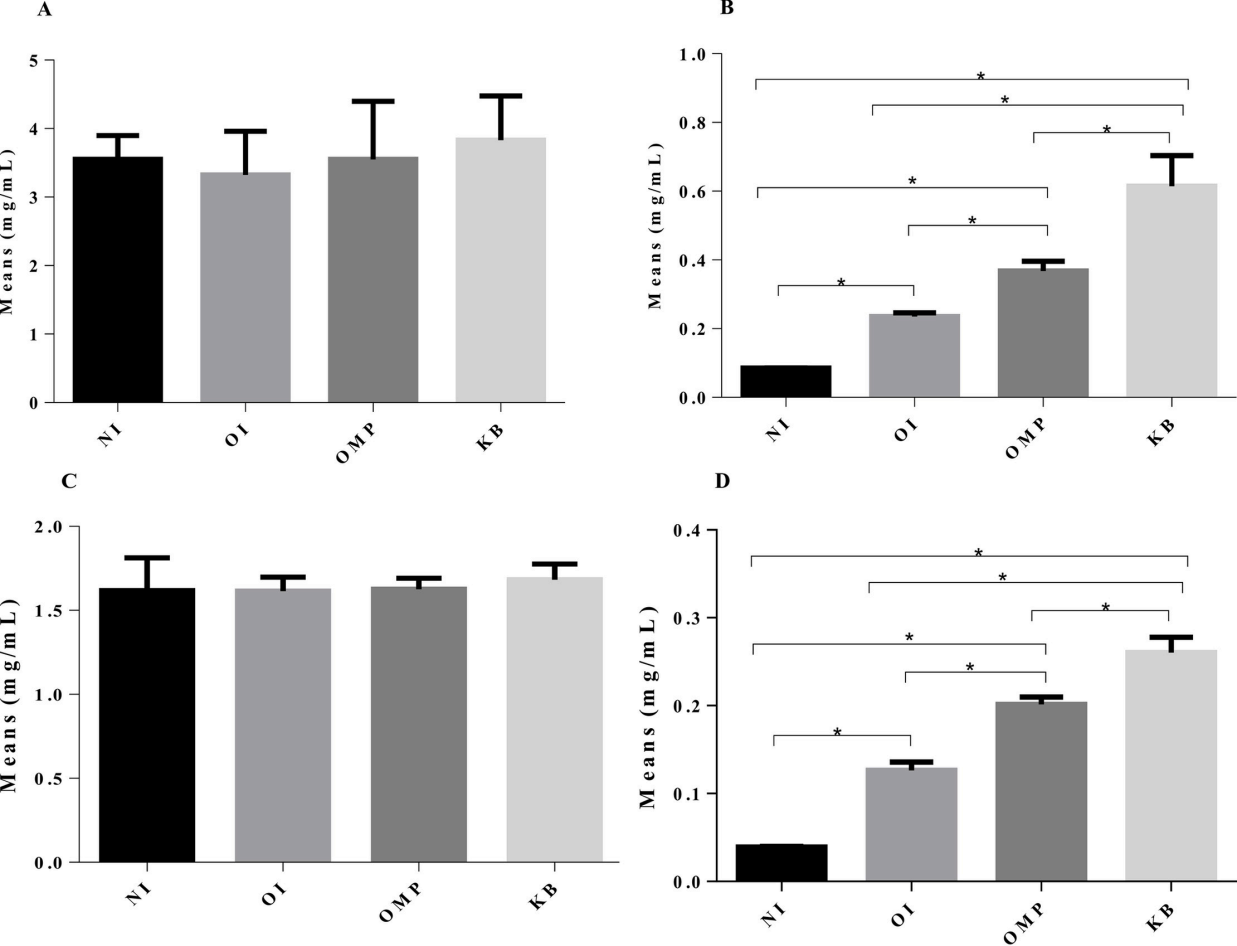
Total and anti-*C*. *jejuni* IgY concentrations in EYP and EEYP. Quantification of IgY from egg yolk powder (EYP) and encapsulated egg yolk powder (EEYP). Fig 1A and 1B represent respectively the concentration of total and anti-*C*. *jejuni* IgYs from EYP. Fig 1C and 1D represent total and anti-*C*. *jejuni* IgYs in EEYP. NI represents the group control not in contact with *C*. *jejuni*, OI represents the group orally challenged with live *C*. *jejuni* mix, OMP represents the group subcutaneously injected with a mix of outer membrane proteins from *C*. *jejuni*, and the KB represents the group subcutaneously injected with a mix of formalin-killed *C*. *jejuni*. horizontal brackets with asterisks refer to significant differences between conditions,* (p <0.05 ANOVA followed by Tukey's post hoc test).

For the EYP, the measured concentrations of total IgY antibodies did not differ whatever the group considered (Fig 1A), but the concentrations of specific anti-*C*. *jejuni* IgYs (Fig 1B) increased significantly depending on the IgY production protocol (0.09 μg/ml for NI, 0.24 μg/ml for OI, 0.37 μg/ml for OMP and 0.61 μg/ml for KB) (Tukey's test, p<0.05). The incorporation ratios of the EYP in the different EEYP reached 44% (NI), 47% (OI), 46% (OMP) and 43% (KB). Concentrations of antibodies were assessed from each EEYP (Fig 1C and 1D) and considering the incorporation ratio values, they did not significantly differ from their corresponding EYP, both for total and anti-C *jejuni* specific antibodies.

### SDS-PAGE and immunoblotting

**Fig 2:**
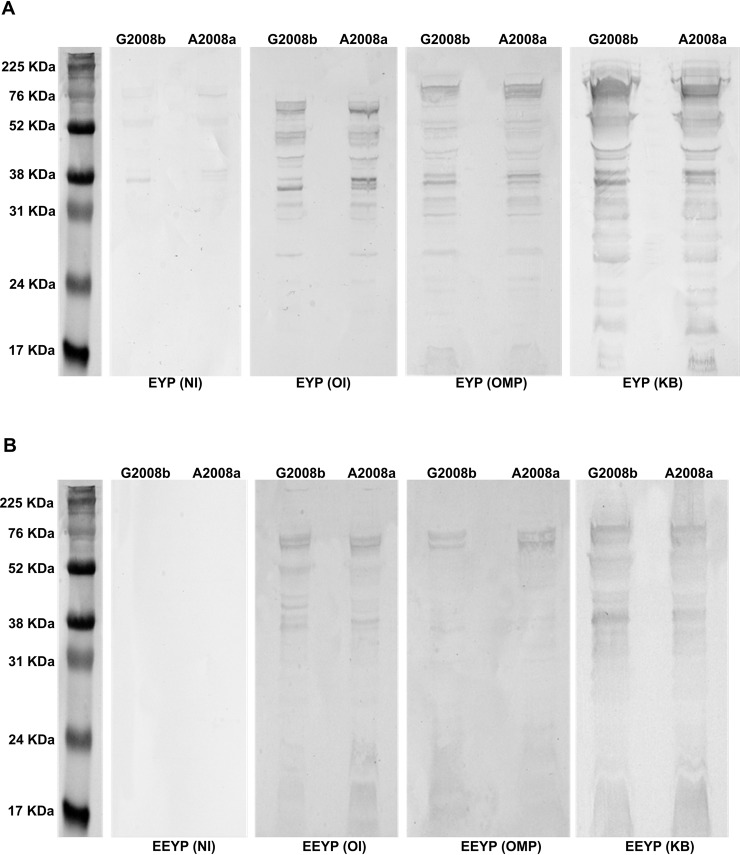
Western blot analysis of recognition of total proteins of *C*. *jejuni* Homologous strains G2008b and A2008a Homologous by EYP and EEY extracts. NI represents the group control not in contact with *C*. *jejuni*, OI represents the group orally challenged with live *C*. *jejuni* mix, OMP represents the group subcutaneously injected with a mix of outer membrane proteins from *C*. *jejuni*, and the KB represents the group subcutaneously injected with a mix of formalin-killed *C*. *jejuni*.

**Fig 3:**
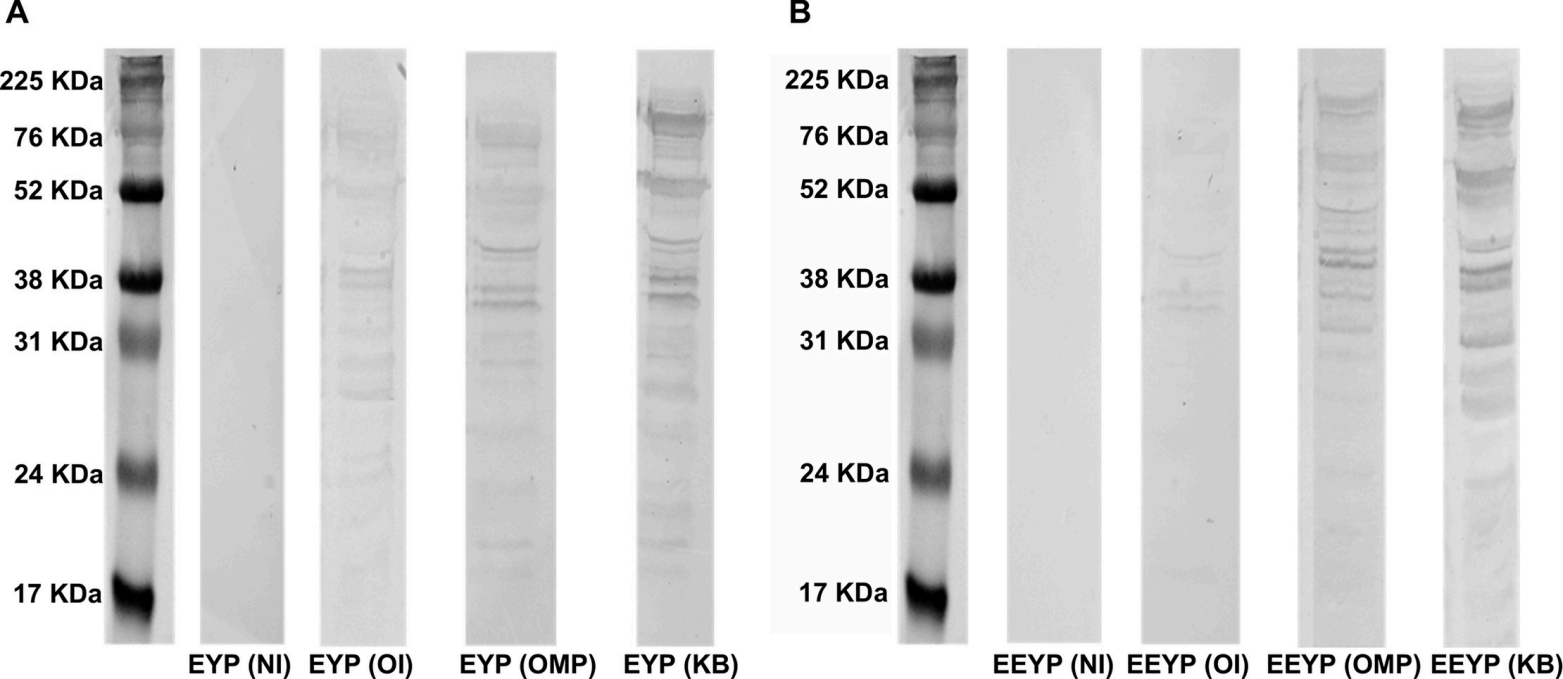
Western blot analysis of recognition of total proteins of *C*. *jejuni* Heterologous strain 81116 by EYP extracts. NI represents the group control not in contact with *C*. *jejuni*, OI represents the group orally challenged with live *C*. *jejuni* mix, OMP represents the group subcutaneously injected with a mix of outer membrane proteins from *C*. *jejuni*, and the KB represents the group subcutaneously injected with a mix of formalin-killed *C*. *jejuni*.

The extracted proteins recognition profiles by the antibody extracted from EYP and EEYP are presented for G2008b and A2008a (Fig 2) as examples of Homologous strains and 81116 (Fig 3) for Heterologuous strains.

Against both Homologous and Heterologous strains, there was virtually no recognition profile with antibodies extracted from control (NI) condition, neither for EYP nor EEYP. On the contrary, IgYs from OI, OMP, and KB provided recognitions profiles with both EYP and EEYP. Total protein profile recognition varied depending on the EYP extracts considered, specifically when extracts came from hens orally inoculated with whole bacteria (OI) compared to IgY extracts recovered after subcutaneous injections with outer membrane proteins (OMP) or formalin killed bacteria (KB). Moreover, the recognition profiles varied in between strains as well as, for a single strain, with the protocol used to produce the eggs. Differences were based on the presence or absence of single bands on the recognition profile.

For the differences according to the egg production protocols, it coherently represented the intensity of seroconversion, increasing when comparing OI versus KB condition and for both injected modes (OMP and KB). As expected, profiles for the extracts originating from the formalin-killed whole bacteria appeared more complete than when only OMP were injected. No indication of a lesser recognition for Heterologous strain could be revealed. Despite the equivalent IgY concentrations for the EYP and EEYP used for these tests, recognition profiles were better defined for the EYP than the EEYP.

### Motility assay

**Fig 4:**
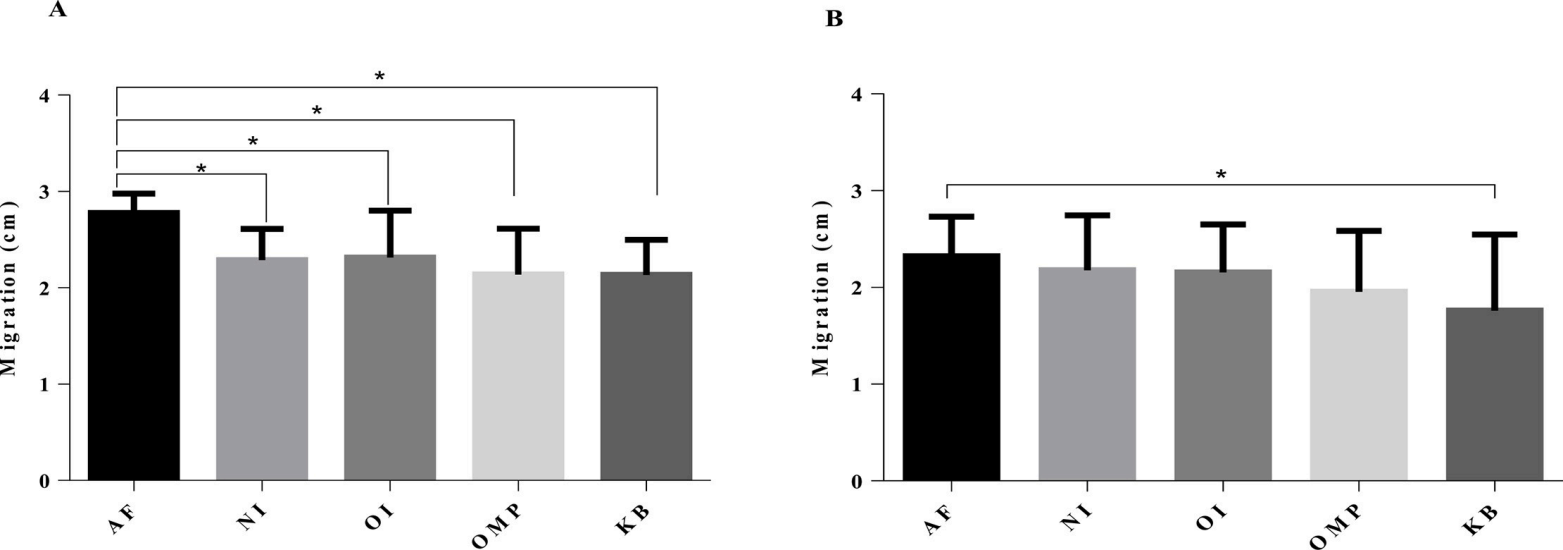
Motility test assessed with or without EYP or EEYP IgY against a mix of Homologous and Heterologous strains. Means of migration in cm of a mix of *C*. *jejuni* Homologous and Heterologous strains, for groups with IgYs compared to no IgYs. NI represents the group control not in contact with *C*. *jejuni*, OI represents the group orally challenged with live *C*. *jejuni* mix, OMP represents the group subcutaneously injected with a mix of outer membrane proteins from *C*. *jejuni*, and the KB represents the group subcutaneously injected with a mix of formalin-killed *C*. *jejuni*. Horizontal brackets with asterisks refer to significant differences between conditions,* (p <0.05 ANOVA followed by Tukey's post hoc test).

A motility test was done to assess the capacity of the EYP extracts to inhibit the motility of *C*. *jejuni*. We observed that whatever the type of egg yolk powder considered, encapsulated or not (EYP and EEYP), all extracts induced a diminution of the strain migration (Fig 4) when compared to the control group without anti-*C*. *jejuni* IgY (AF). A significant lower motility was recorded for groups NI, OI, OMP and KB when using the EYP (Fig 4A) but the effect was significant only for the KB group when EEYP extracts were tested (Fig 4B).

When analyzed at the individual strain level (Tables 2 and 3), the motility reduction appeared not only dependent of IgY production protocol but also strain dependent. For example, the EYP issued from the protocol using OMP significantly limit the migration of four out all seven individually tested strains (Homologous and Heterologous). The same test with KB limit motility for all seven strains tested. The different groups of antibodies were strain-dependent and IgY production protocol dependent for both EYP and EEYP (Table 3).

**Table 2:** Means of migration diameters of each strain in presence of EYP IgYs.

	EYP			
	NI	OI	OMP	KB
**G2008b**	**2.67 ± 0.12**	2.07 ± 0.06*	2.00 ± 0.12*	1.77 ± 0.00*
**A2008a**	**2.87 ± 0.12**	**2.63 ± 0.00**	**2.70 ± 0.12**	2.37 ± 0.15*
**B2008c**	**3.00 ± 0.00**	2.50 ± 0.00*	**2.70 ± 0.29**	2.67 ± 0.00*
**RM1221**	**2.67 ± 0.29**	**1.83 ± 0.25**	1.43 ± 0.15*	1.37 ± 0.00*
**ATCC 700819**	**2.80 ± 0.06**	2.37 ± 0.00*	2.40 ± 0.00*	2.40 ± 0.06*
**81116**	**2.77 ± 0.35**	**2.37 ± 0.12**	**2.63 ± 0.25**	2.27 ± 0.25*

Mean diameter of migration zone in cm +/- SD; n = triplicate

* = significant difference with NI; NI represents the group control not in contact with *C*. *jejuni*, OI represents the group orally challenged with live *C*. *jejuni* mix, OMP represents the group subcutaneously injected with a mix of outer membrane proteins from *C*. *jejuni*, and the KB represents the group subcutaneously injected with a mix of formalin-killed *C*. *jejuni*.

**Table 3:** Means of migration diameters of each strain in presence of EEYP IgYs.

	EEYP			
	NI	OI	OMP	KB
**G2008b**	**2.67 ± 1.15**	**2.67 ± 0.29**	**2.50 ± 0.25**	**2.77 ± 1.08**
**A2008a**	**2.53 ± 0.06**	**2.50 ± 0.00**	**2.50 ± 0.00**	2.40 ± 0.00*
**B2008c**	**1.53 ± 0.06**	**1.50 ± 0.00**	1.20 ± 0.00*	1.33 ± 0.06*
**RM1221**	**1.93 ± 0.12**	**1.67 ± 0.29**	1.13 ± 0.12*	1.07 ± 0.12*
**ATCC 700819**	**2 ± 0.00**	**2 ± 0.00**	2 ± 0.00*	1 ± 0.00*
**81116**	**2.40 ± 0.00**	**2.60 ± 0.17**	**2.40 ± 0.17**	2.00 ± 0.00*

Mean diameter of migration zone in cm +/- SD; n = triplicate

* = significant difference with NI. NI represents the group control not in contact with *C*. *jejuni*, OI represents the group orally challenged with live *C*. *jejuni* mix, OMP represents the group subcutaneously injected with a mix of outer membrane proteins from *C*. *jejuni*, and the KB represents the group subcutaneously injected with a mix of formalin-killed *C*. *jejuni*.

### Bactericidal assay

The test was used to appreciate the capability of the EYP to kill *C*. *jejuni* in the presence of serum (complement). The *Campylobacter* counts in the presence of complement were compared to those in the presence of inactivated complement only for the groups OMP and KB which contained the highest concentrations of antibodies. Although the values obtained seemed to indicate a reduction in *C*. *jejuni* viability (S1 Fig), it did not achieve the level of statistical significance.

### *In vivo* assay

**Fig 5:**
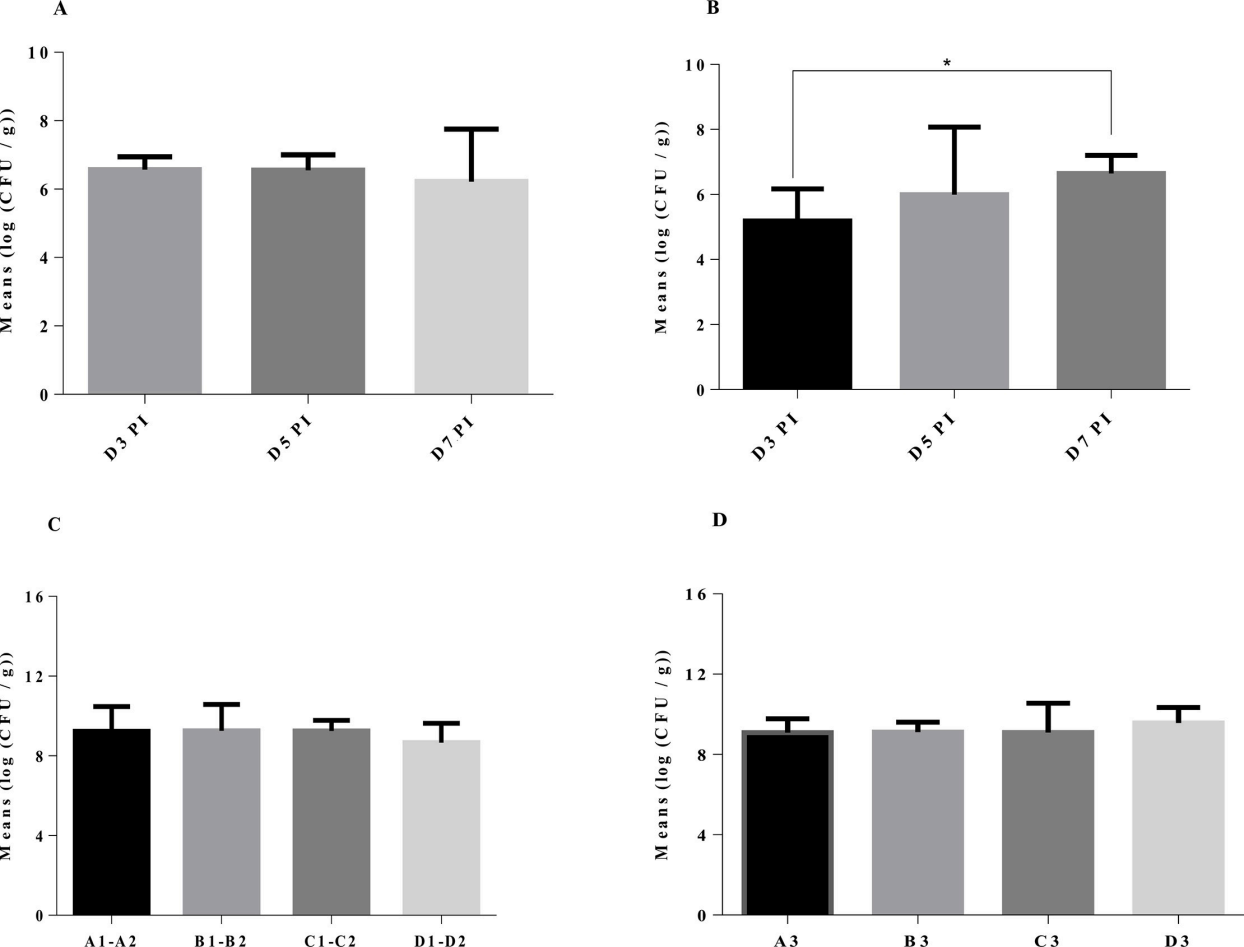
*C*. *jejuni* counts in excreted feces and cecal contents. EEYP was incorporated at 5% in the feed. Fig 5A and 5B represent the level of *C*. *jejuni* from excreted fecal matter from D3 to D7 PI. Fig 5C and 5D represent the colonization levels in caecum D7 PI. A1, A2, B1, B2, C1, C2, D1 and D2 were in the same room and were inoculated with Homologous strains (G2008b, A2008a, B2008b and RM1221). A1 and A2 received only encapsulated matrix. B1 and B2 received NI EEYP. C1 and C2 received OI EEYP. D1 and D2 received a mix of OMP and KB EEYP. A3, B3, C3, and D3 were in the same room and were inoculated with Heterologous strains (ATCC 700819, 81116). A3 received only encapsulated matrix. B3 received NI EEYP. C3 received OI EEYP and D3 received a mix of OMP and Gr KB EEYP. Horizontal brackets with asterisks refer to significant differences between conditions,* (p <0.05 ANOVA followed by Tukey's post hoc test).

To evaluate the capacity of the produced EYP to protect birds from *C*. *jejuni*, an *in vivo* assay was conducted. It compared three types of encapsulated EEYP: not potentiated against *C*. *jejuni* (NI), colonization of the birds after oral inoculation with live bacteria (OI), and a mix from the OMP and KB groups which presented almost equivalent quantity and quality of their characterized anti *C*. *jejuni* antibodies (Fig 5).

Despite receiving the EEYP for 12 days, all groups orally inoculated with a light inoculum (10^3^ CFU/bird) of *C*. *jejuni* Homologous or Heterologous strains, were colonized by *Campylobacter* as soon as day three post-inoculation, as demonstrated by mean excreted counts of 7 and 5 log UFC/g for Homologous and Heterologous groups respectively (Fig 5A and 5B). Excretion of *C*. *jejuni* was rapid and stable for Homologous group (Fig 5A) while colonization was more progressive for Heterologous group (Fig 5B). Caecal colonization (D7 PI) reached between 8 and 10 log CFU *C*. *jejuni* /g of contents for all groups of birds (Fig 5C and 5D). EEYP did not prevent nor reduced colonization of the birds’ caeca by *C*. *jejuni*. Mean and standard deviation of total IgY concentration in ng/g of caecal matter was: control = 37.85 +/- 26.47; NI = 39.54 +/- 26.47; OI = 45.42 +/- 32.12; OMP + KB = 60.87 +/- 34.03. No differences (p = 0.06) were observed between chicken groups.

## Discussion

Our results indicate that the different modes of IgY production are not equivalent in their ability to produce eggs powders with anti-*C*. *jejuni* activity. First In a quantitative perspective: anti-*C*. *jejuni* IgYs concentrations in OMP (subcutaneously injected with *C*. *jejuni* mix outer membrane proteins) or KB (subcutaneously injected with formalin-killed *C*. *jejuni* mix) derived EYP and EEYP were significantly higher than a *per os* inoculation (OI) (orally challenged with live *C*. *jejuni* mix) derived EYP or EEYP. Similar results were obtained for the egg yolks prior to transformation to EYP and subsequently to EEYP [23].

Moreover, in a qualitative perspective, *in vitro* results show that both EYP and EEYP IgYs in OI, OMP, and KB could react with both Homologous and Heterologous *C*. *jejuni* strains, confirming that inoculation of laying hens with OMP, killed or live bacteria can induce functional antibodies against *the bacterium* and these properties are retained following transformation of the egg yolks [23] into EYP and EEYP.

The fact that the antibodies of the OI could recognize the different strains of *C*. *jejuni* in this study means that natural contamination of birds, resulting in a gastrointestinal colonization by *C*. *jejuni*, is able to induce a seroconversion [27] sufficient to be translated to the egg yolk powders. Our work using different IgY production modes proved that it is possible to increase the quantity of anti-*C*. *jejuni* antibodies in EYP. The *in vitro* results also show that antibodies extracted from the different groups could all provide a broad spectrum of *C*. *jejuni* protein recognition for several different *C*. *jejuni* strains. OMP and KB derived EYP or EEYP appeared to be able to provide greater protection, considering they showed a stronger recognition and a greater number of recognized *C*. *jejuni* proteins.

The motility assays showed that whatever the type of egg yolk powder considered, *i*.*e*. encapsulated or not, and for the several inoculation modes, *C*. *jejuni* reduce motility occurred and was strain dependent. Moreover it is interesting to notice that all 4 groups induced a diminution of the motility of *Campylobacter* when compared to a condition without egg yolk derived antibodies (AF). The mobility tests confirm that unidentified elements, present in non-immunized egg extracts (NI), are sufficient to perturb *C*. *jejuni* motility *in vitro* which is an important function of *C*. *jejuni* pathogenesis [28]. Moreover, the NI extracts were not able to induce agglutination or reveal any western blot recognition profile. The observed impairment of *C*. *jejuni* motility in the NI extracts therefore does not appear related to the presence of antibodies in the egg yolk. This is in accordance with previous studies that showed, under some condition, the ability of generic eggs to control, to some extent, pathogens presence in chickens [29, 30].

In *vitro*, no bactericidal effect was observed for EYP containing the highest concentrations of antibodies (OMP and KB) in presence of complement although in other studies, *C*. *jejuni* has been shown to be susceptible to killing by maternal antibodies, the killing being mediated by both complement and specific antibody [31]. It is unclear what contributed to the incapacity to these EYP *in vitro* to kill the strains used in this study but a preliminary digestion step to release antibodies from the powders could be required.

Al-Adwani et al. also produced well characterized hyper-immunized EYP but against a limited number of targeted outer membrane proteins of *C*. *jejuni* [26]. EYP also failed to demonstrate an effect against *C*. *jejuni* colonization in poultry despite their use at 10% in the feed [20]. This raised the questions of the ability of the antibodies to reach an efficient intestinal location, and the usefulness of a larger *C*. *jejuni* antigen recognition profile, as suggested by Yeh et al [32].

Our microencapsulation of the egg yolk powder preserved quantities and in *in vitro* efficiency against important functions of *C jejuni* such as mobility and agglutination, both recognized as virulence associated properties, but with a reduced magnitude. We therefore considered the opportunity of testing these EEYP as feed additives for the reduction of the chicken colonization by *C*. *jejuni* as we hypothesized that the powders should be protected from digestive degradation by the encapsulation process.

Our data demonstrated that antibodies were delivered in the caecum of birds. Despite this and using an inoculation model that lightly challenged the birds (inoculum of 10^3^ CFU per bird), the EEYP treatment did not prevent rapid colonization, as demonstrated by fecal excretion, nor limit the level of *C*. *jejuni* in the digestive tracts of the birds.

Our *in vivo* results confirm and complete the work of Paul et al [20] whose results suggested that a long-term prophylactic treatment (until 3 weeks of age) with 5% of hyper-immunized egg yolk powder or non-immunized egg yolk powder in feed was not sufficient to significantly reduce caecal counts of *C*. *jejuni*. Interestingly, Kassaify and Mine [18] previously reported that prophylactic treatment with 10% non-immunized egg yolk powder for 4 weeks before infection resulted 3 to 4 log reduction of fecal *C*. *jejuni* counts in laying hens of 22–24 weeks of age. However, before attributing these results to the non-potentiated EYP, it is important to establish the status of hens providing eggs for this trial, because chicken colonization by *Campylobacter* is frequent [33] and colonized chickens can yield eggs with anti-*C*. *jejuni* antibodies as observed in our present study.

Ours results appears also in contradiction with those of Hermans et al., [19] who suggested that the use of an EYP potentiated against the hydrophobic protein fraction of one *C*. *jejuni* strain, incorporated at 5% in the diet for 3 days before infection, could block *C*. *jejuni* transmission and lead to a drastic reduction of colonization of the same *C*. *jejuni* strain. In commercial conditions, colonization generally appears after the first 2–3 weeks of age, although it is possible experimentally in chicks of one day old. This lag phase might be explained by the digestive microbiota of the young chick that would prevent colonization by *C*. *jejuni* [34]. Although our results are in contrast with those of Hermans [19], it is important to notice that in our work, birds were treated for 2 weeks before infection and received a maximum EEYP incorporated in their feed (5%). The EYP that contained specific antibodies against *C*. *jejuni* was 45% (w/w) encapsulated in a lipidic matrix. Taking into account the incorporation ratios, we can consider that the effective concentration was about 2.5%, which was lower than what was used in other studies, but that should have been protected against degradation by the digestive process. Moreover, nonparametric analyzes of cecal antibodies concentration between groups indicate a trend (p = 0.06) that the OMP/KB group regularly presented more caecal IgY, supporting a protective effect of the encapsulation, despite a high inter-individual variability. Although the powders/IgY appear to have been released, they have not been effective in modulating the bird’s colonization by *C*. *jejuni*.

Our inoculum contained several *C*. *jejuni* strains known to be able to colonize chickens to a high level [35] therefore representing a greater challenge for the EEYP effectiveness. Within commercial broiler flocks, multiple strains of *C*. *jejuni* may colonize [36] and it is well known that there is variability in the capacity of colonization and virulence among different *C*. *jejuni* strains [37]. Chicken *C*. *jejuni* strains harbor genetic and phenotypic diversity [38] and it was shown that when co-inoculated, some *C*. *jejuni* strains compete to colonize the chicken intestine and that strain could dominate others [39]. Given the strain-dependent effect we observed with the EYP and EEYP derived IgY characterization, this phenomenon may have taken place in our study and may have counterbalanced the protective effect that could have been brought by the use of the EEYP. This colonization competition and strain-dependent efficiency of EYP could also be at play in other studies that used less characterized strains or single strains in their *in vivo* trials. Therefore, in these studies, it hardly allows the comparison of the effectiveness of the egg yolks powders when confronted to a broader diversity of *C*. *jejuni* strains. Hence our infection model represents a greater challenge for an in feed additive candidate but seems to better represent the poultry production reality.

The divergence between our results and those published in the scientific literature in assessing the efficiency of using egg yolk powder as an additive to prevent *C*. *jejuni* colonization in poultry suggests that the optimal conditions for their use in field conditions have yet to be fully identified. Our work also indicates that absence of efficiency does not appear to be due to a loss of quantity or functionality of the antibodies after encapsulation.

## Supporting information

S1 Fig The bar represents the mean count (n = 8) of still cultivable bacteria in the presence of complement (Test) or inactivated complement (Control) in presence of EYP containing the highest concentrations of antibodies against OMP and KB. (DOCX)
