# Correction: *In vitro* efficacy of potentiated egg yolk powder against *Campylobacter jejuni* does not correlate with *in vitro* efficacy

**DOI:** 10.1371/journal.pone.0215699

**Published:** 2019-04-16

**Authors:** Amina Soumaila Garba, Alexandre Thibodeau, Audrey Perron, Sylvette Laurent-Lewandowski, Ann Letellier, Philippe Fravalo

In the title of the article, the second “in vitro” should be “in vivo.” The correct title is: *In vitro* efficacy of potentiated egg yolk powder against *Campylobacter jejuni* does not correlate with *in vivo* efficacy.
