# Supplementary material for: In vitro efficacy of potentiated egg yolk powder against Campylobacter jejuni does not correlate with in vitro efficacy
Source: PLoS One. 2019 Mar 7;14(3):e0212946. doi: 10.1371/journal.pone.0212946 (PMC6405129; doi:10.1371/journal.pone.0212946)
Supplement: S1 Fig — (DOCX) [file pone.0212946.s001.docx]

S1 Fig

*The bar represents the mean count (n=8) of still cultivable bacteria in the presence of complement (Test) or inactivated complement (Control) in presence of EYP containing the highest concentrations of antibodies against OMP and KB.*
